# Dysphagia Caused by Spindle Cell Lipoma of Hypopharynx: Presentation of Clinical Case and Literature Review

**DOI:** 10.1155/2012/107383

**Published:** 2012-12-26

**Authors:** Alberto Peña-Valenzuela, Nathalia García León

**Affiliations:** Otolaryngology Department, Universidad Nacional de Colombia, Carrera 35A No. 57-91, Apartamento 304, Bogotá, Colombia

## Abstract

Spindle cell lipoma of the hypopharynx is an extremely rare entity. Here, we present the first case of this lesion originated in the cricopharyngeal region, with symptoms of chronic progressive dysphagia, which can be confused with other pathologies; endoscopic and magnetic resonance imaging (MRI) evaluation are the methods of choice for its diagnostic approach. The best therapeutic approach is endoscopic resection with rapid recovery and few complications. Long-term followup is recommended, either endoscopic or imaging, given that it can be confused with an undiagnosed liposarcoma; additionally, its long-term behavior is unknown.

## 1. Introduction

Lipomas are mesenchymal tumors derived from mature adipocytes, characterized by their slow growth; in pharynx and hypopharynx, they have been reported as extremely rare, being 0.6% of all benign neoplasms, with a total of 80 cases reported in that location [1]. They are originated from normal adipose tissue adjacent to the hypopharynx, and can be sessile or pedunculated, well encapsulated, and of smooth consistency. Due to their slow growth and difficult location, they can be confused with other pathologies. 

The histological variety of spindle cell lipoma is quite uncommon; being 1.5% of all lipomas, it is a tumor derived from prelipoblastic mesenchymal cells, pathogenesis is unknown, but recently cytogenetic changes have been found characterized by monosomy or partial loss of chromosome 13 and/or 16 [2]. 

## 2. Case Report

**Figure 1:**
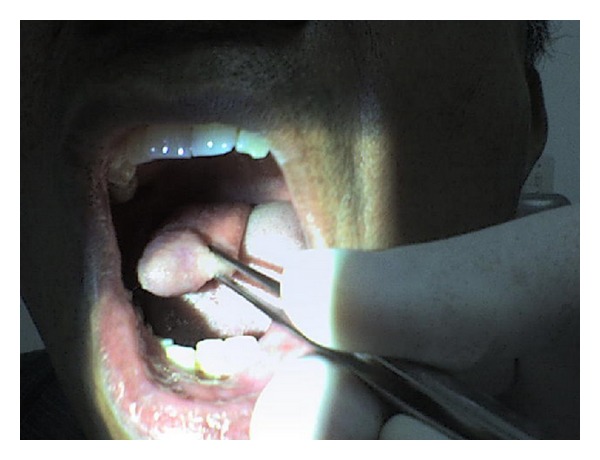
Protrusion of the mass with Valsalva.

We present the case of a 66-year-old male patient with a condition of progressive dysphagia of five-year evolution, who two years ago presented a protrusion of mass in the oral cavity provoked by Valsalva maneuver or intra-abdominal pressure increase. The patient was referred to our institution with diagnosis of Zenker's diverticulum. During the first consultation, the protrusion of a well-defined elongated mass was confirmed via oral cavity with the Valsalva maneuver (Figure 1). A barium esophagogram was conducted without revealing the presence of esophageal diverticulum; contrasted neck CT scan showed no abnormalities or presence of masses. Upper gastrointestinal endoscopy evidenced partial occupation of the esophagus by an elongated mass, with soft consistency, approximately 16 to 23 cm from the dental arch. 

**Figure 2:**
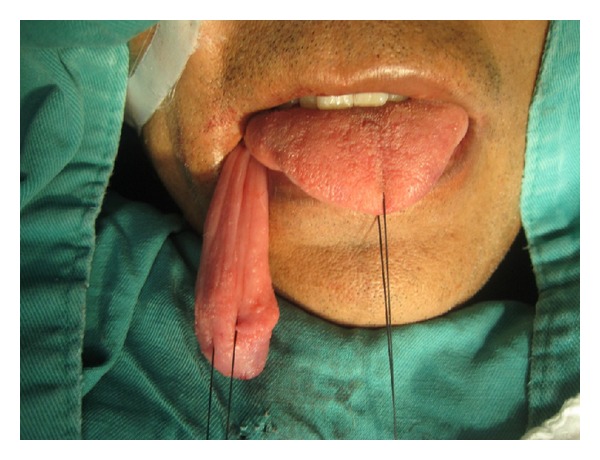
Intraoperative image.

**Figure 3:**
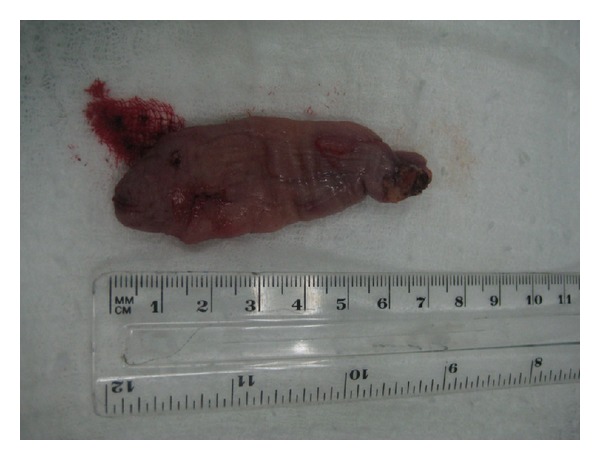
Excised lesion.

With the previous findings, it was decided to bring the patient to surgery for identification and extraction of the mass. Under general anesthesia, intraoperative esophagoscopy was performed with identification of the pedunculated mass dependent on the left cricopharyngeal region and extrusion by means of an endoscopic loop; the lesion was resected from its pedicle with harmonic scalpel (Figures 2 and 3).

**Figure 4:**
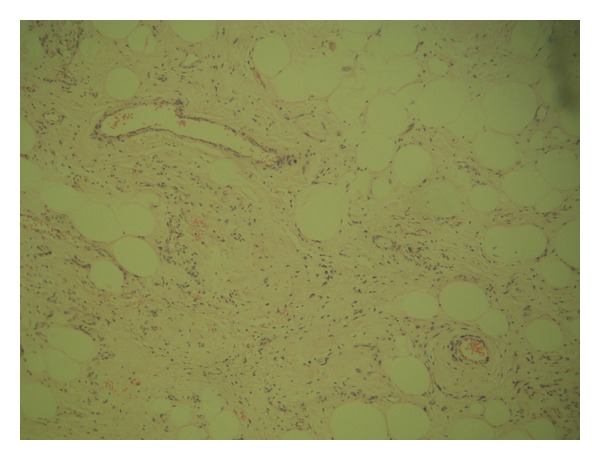
Lesion stained with H/E.

**Figure 5:**
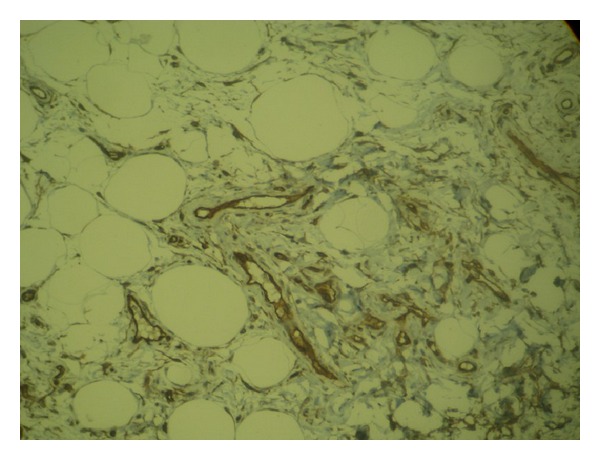
CD34 reactivity.

The histological analysis of the lesion revealed a 6 × 2.5 × 1.5 mm dependent soft-tissue, polypoid circumscribed lesion, lined by stratified squamous epithelium without dysplasia, constituted by spindle cells without atypia and mature adipose tissue, without observing atypical mitosis (Figure 4). The immunohistochemical profile revealed positivity of spindle cells for CD34, negativity for S100, and a low proliferation with Ki57, which confirmed the diagnosis of spindle cell lipoma and revealed its benign behavior (Figure 5). 

The patient had satisfactory postoperatory evolution, with initiation of diet on the second day and discharged on the third day, without respiratory distress, dysphagia, or any other symptomatology. Endoscopic control was conducted one week later with evidence of a healthy scarred stump. Upon control one year after the procedure no lesion recurrence was registered.

## 3. Discussion

Spindle cell lipoma originated from the hypopharynx is an extremely rare entity, with the finding of four cases reported in the literature in that location. We present a case of spindle cell lipoma with symptomatology of long-standing dysphagia and as single data, with protrusion of such with the Valsalva maneuver, initially confused with Zenker's diverticulum. 

Macroscopically, these are sessile or pediculated masses of soft textures and well circumscription, which can be confused with other benign lesions, as in the case introduced. According to existing reports, these are described as slow-growing solitary masses, which originated in the hypopharynx and can present symptoms like dysphagia, dysphonia, stridor, foreign body sensation, or long-standing history of nonspecific symptoms [3]; cases have been reported with sudden stridor [4] and one case with sudden death caused by asphyxiation secondary to a hypopharyngeal lipoma [5]. 

Among the histological variants of lipomas, there are angiolipoma, angiomyolipoma, pleomorphic, benign lipoblastoma, fibrolipoma, chondrolipoma, and spindle cell lipomas; this last variety is characterized by presenting abundant mature adipocytes on a collagenous matrix with spindle cells and variable vascular patterns. They are differentiated from liposarcomas by the cellular uniformity and lack of lipoblasts and of cellular pleomorphism. Spindle cells are similar to fibroblasts, with elongated nucleus, which react to CD34. The adipocytes present in the spindle cell lipoma are of mature characteristics and although classically fat cells are S100-reactive, in this histological subtype reactivity is not present [6]. 

Among the diagnostic means described for the assessment of the lesion, one of the fundamental pillars is the endoscopic visualization, finding a single pediculated lesion, coated with mucosa of normal appearance, of soft texture, and well circumscription, which in the case presented is inserted in the cricopharyngeal region. Although in this case the contrasted neck CT scan did not contribute much information, cases published report that means of diagnosis may offer an approximation of the extension of the lesion and reveal if there is or is not infiltrative behavior toward deep tissue, as well as the presence of lymphadenopathy suspicious of malignity; however, upon treating a dependent soft tissue lesion, the MRI has better definition in the evaluation of soft tissue and its extension to adjacent structures and should be considered if doubts emerge regarding the lesion [7]. 

Treatment of hypopharynx spindle cell lipoma consists in the radical excision of the lesion, ideally under endoscopic vision, given that because of its nature and location with this approach its complete resection is possible, with rapid recovery not exceeding three days, as shown in the case presented, without report to date of complications with this method [8, 9]. Endoscopic followup of these lesions is recommended; despite followup at 18 months of the cases reported, no recurrence has been found of such, and their long-term behavior is yet unknown.
